# Effects of depth of neuromuscular block on surgical operating conditions in women undergoing gynecologic laparoscopic surgery: a randomized clinical trial

**DOI:** 10.1186/s44158-023-00086-7

**Published:** 2023-01-19

**Authors:** Antonio Coviello, Marilena Ianniello, Pasquale Buonanno, Nausica Di Falco, Carmine Iacovazzo, Alfredo Maresca, Maria Vargas, Annachiara Marra, Agostino Candice, Gabriele Saccone, Fulvio Zullo, Giuseppe Servillo

**Affiliations:** Department of Neuroscience Reproductive Science and Dentistry, School of Medicine, Federico II University, Naples, Italy

**Keywords:** Gynecologic surgery, Laparoscopic surgery, Neuromuscular block, Clinical trial, Post-anesthesia care unit, PACU, Surgical conditions

## Abstract

**Background:**

In recent years, the relationship between the advantages and disadvantages of a deep neuromuscular block (DNMB), compared to a moderate block (MNMB) in laparoscopic surgery, has been increasingly studied.

**Objective:**

Evaluate the effect of D-NMB compared to M-NMB in gynecological laparoscopic surgery.

**Methods:**

This was a parallel-group, double-blind, randomized clinical trial, conducted at a single center in Italy between February 2020 and July 2020. American Society of Anesthesiologist (ASA) I–II risk class patients scheduled for elective gynecological laparoscopic surgery were randomized into a 1:1 ratio to either experimental or control group. The first one included DNMB with a rocuronium bolus at the starting dose of 1.2 mg/kg and a maintenance dose (0.3–0.6 mg/kg/h). The second one included MNMB with a rocuronium bolus at the starting dose of 0.6 mg/kg, and a maintenance dose in boluses (0.15–0.25 mg/kg). The primary outcome was the intraoperative surgical condition assessed every 15 min by the surgeon as a 5-point scale. The secondary outcome was the time needed to discharge patients from post-anesthesia care unit (PACU). The tertiary outcome was the assessment of the intra-operative hemodynamic instability. A sample size of 50 patients was planned.

**Results:**

One hundred five patients were assessed for eligibility, 55 were excluded. Fifty patients met the inclusion criteria and were enrolled. The average score for the operative field was 4 for the D-NMB group and 3 for the M-NMB group (*p* value < 0.01). The length of stay in PACU was 13 min for the DNMB group and 22 min for the MNMB group (*p* value = 0.02).

**Conclusions:**

Deep neuromuscular block improves intraoperative surgical condition during gynecological laparoscopic surgery.

**Trial registration:**

clinicalTrials.gov NCT03441828

## Introduction

Laparoscopy represents one of the most used surgical techniques in many fields. Compared to open surgery, laparoscopy has many advantages, including less blood leak [1, 2], early recovery of recanalization, mobilization [3] and personal care, reduced length of stay in the hospital, less risk of infections and less demand for analgesics [4].

Moreover, the European Association for Endoscopic Surgery (E.A.E.S.) suggests that the lowest intra-abdominal pressure (IAP), necessary to obtain a suitable operating field, has to be used. Deep neuromuscular block (D-NMB) may allow the creation of a low-pressure pneumoperitoneum without any negative impact on the operative field and operative time. However, the effect of D-NMB compared to moderate neuromuscular block (M-NMB) in gynecological laparoscopic surgery is still a subject of debate [5, 6]

Thus, the aim of this study was to evaluate the effect of D-NMB versus M-NMB in gynecological laparoscopic surgery.

## Methods

### Study design

This was a single center, parallel-group, double-blind, randomized controlled trial, conducted between February 2020 and July 2020 at the University of Naples “Federico II”, in Naples, Italy. The trial was approved by the local ethics committee. All participants in the trial provided written informed consent. Surgeons, patients and outcomes’ assessors were blinded to the group assignment. The surgical team that performed the procedures was the same for both groups. All data were collected by anesthesia residents involved in the research.

Inclusion criteria included: age between 18 and 60 years; American Society of Anesthesiologist (ASA) risk class I–II; elective laparoscopic surgery with an expected duration of 60 min or less. Exclusion criteria included: body mass index (BMI) > 30; liver or kidney dysfunction; neuromuscular disorders; hypersensitivity to drugs used as per protocol; pregnancy. Patients with conversion to open laparotomy or operative time longer than 60 min were excluded.

Experimental group received a D-NMB through a starting bolus of rocuronium at a dose of 1.2 mg/kg, and a continuous titrated infusion (0.3–0.6 mg/kg/h) to maintain a train of four (TOF) count of 0, and a post-tetanus count (PTC) between 1 and 2.

Control group received a M-NMB through a single starting bolus of rocuronium at a dose of 0.6 mg/kg and administering additional boluses (0.15–0.25 mg/kg) to maintain a TOF count of 1–3, if needed.

During surgery, intraperitoneal insufflation pressure was initially set to 9 mmHg and then was modified as surgeon required.

Intraoperative surgical conditions were assessed by surgeon as 5-point Likert scale score:

1 point—extremely poor or the surgeon is unable to obtain a visible laparoscopic field due to inadequate muscle relaxation, therefore additional NMBAs is given; 2 points—poor or there is a visible laparoscopic field, but the surgeon is very hampered by inadequate muscle relaxation with continuous muscle contractions and hazard of tissue damage, therefore additional NMBAs are given; 3 points—acceptable or there is a wide visible laparoscopic field but muscle contractions occur regularly causing some interference with the surgeon’s work, therefore additional NMBAs to prevent damages are necessary; 4 points—good or there is a wide laparoscopic working field with sporadic muscle contractions, therefore additional NMBAs are not immediately necessary; 5 points—Optimal or there is a wide visible laparoscopic working field without any muscle contraction, therefore additional NMBAs are not necessary.

### Randomization and blinding

Patients enrolled were assigned to the D-NMB or M-NMB group in a 1:1 ratio by a web-based system using no random blocks (www.randomizer.org/form.html).

Patients, surgical and anesthesia teams, and postoperative caregivers were blinded for treatment allocation. An unblinded expert researcher ensured adherence to the trial protocol during the procedures. A syringe pump with rocuronium was prepared for every patient, regardless of the treatment allocation, to avoid unblinding of the teams. The rocuronium syringe and the neuromuscular monitor were covered in such a way that medical teams, in the operating room, were not able to read infusion rates or NMB depth.

Likert scale was done by the blinded surgeon and the anesthesiologist; the unblinded researcher was not involved in the Likert scale. The investigators who assessed postoperative secondary endpoints or perform final data analysis were blinded to the group allocation. Participating patients were blinded until study completion.

After the Likert scale was obtained by both the surgeon and anesthesiologist, the anesthesiologist was unblinded regarding the depth of NMB to ensure adequate NMB reversal. The unblinded researcher administered additional rocuronium if the surgery required administration of additional muscle relaxants. These procedures assured that unblinding was not needed during the procedure. The surgeon remained blinded also in this event.

### Sample size calculator

The sample size calculation was based on previous studies (i.e., according to Martini‘s et al. “Evaluation of surgical conditions during laparoscopic surgery in patients with moderate vs deep neuromuscular block” [6] and according to Williams’ et al. “A comparison of the effect of two anesthetic techniques on surgical conditions during gynecological laparoscopy”^7^).

### Strategies adopted in case of intra-operative hypotension

Hypotension was identified as a mean arterial pressure (MAP) lower than 60 mmHg or a systolic arterial pressure (SAP) lower than 90 mmHg or a drop in SAP of 20% compared to pre-induction values.

Treatment administered to control intra-operative hypotension was ephedrine at a dose of 0.1 mg/kg iv when associated to bradycardia or phenylephrine at a dose of 100 mcg/each bolus iv when associated to tachycardia.

### Outcomes

Intraoperative surgical conditions assessed every 15 min by surgeon as 5-point Likert scale score was the primary outcome. The secondary outcomes were time to discharge from post-anesthesia care unit (PACU) to inpatient bed; mean intraperitoneal insufflation; necessity of Trendelenburg position; peak airway pressure after CO2 insufflation; intraoperative anesthetic and surgical complications; PONV; postoperative pain; respiratory complications. Assessment of intra-operative hemodynamic instability defined as MAP lower than 60 mmHg or a systolic arterial pressure (SAP) lower than 90 mmHg or a drop in SAP of 20% compared to pre-induction values was the tertiary outcome

### Anesthesia protocol

One hour before surgery, all patients received 75 mg of subcutaneous injection of diclofenac. Pantoprazole 40 mg iv and ondansetron 4 mg iv were administered as premedication.

Standard monitoring was applied according to local protocols including electrocardiography, pulse oximetry, and non-invasive blood pressure.

Anesthetic depth was monitored using a bi-spectral index (BIS) monitor (Aspect A-20001, Aspect Medical system Inc., Newton, MA, USA).

Neuromuscular monitoring was performed by applying TOF-Watch device ((MSD, Haarlem, The Netherlands) to patient’s arm.

Balanced anesthesia was inducted with propofol (2 mg/kg) and fentanyl (4 mcg/kg) and was maintained administering sevoflurane at a minimum alveolar concentration (MAC) of approximately 0.9.

Tracheal intubation was performed after administering a bolus of rocuronium bromide as per the randomization protocol. Mechanical ventilation was adjusted to maintain End-tidal CO_2_ between 30 and 35 mmHg.

The target level of the BIS was between 40 and 60 during the surgery. PTC was evaluated every 5 min.

After the insertion of Verres needle, pneumoperitoneum was obtained with a pressure of 15 mmHg and then lowered to 9 mmHg. In case of insufficient or inadequate surgical condition, intrabdominal pressure insufflation was increased to 12 mmHg in both groups. Additional measures were additional bolus of rocuronium in M-NMB group and titration of the continuous infusion of rocuronium in D-NMB group.

Sugammadex, at a dose of 4 or 2 mg/kg iv, was administered as neuromuscular reversal to D-NMB and M-NMB group, respectively.

Extubation was performed at a TOF ratio of 0.9 and then the patients were transferred to the PACU.

**Fig. 1:**
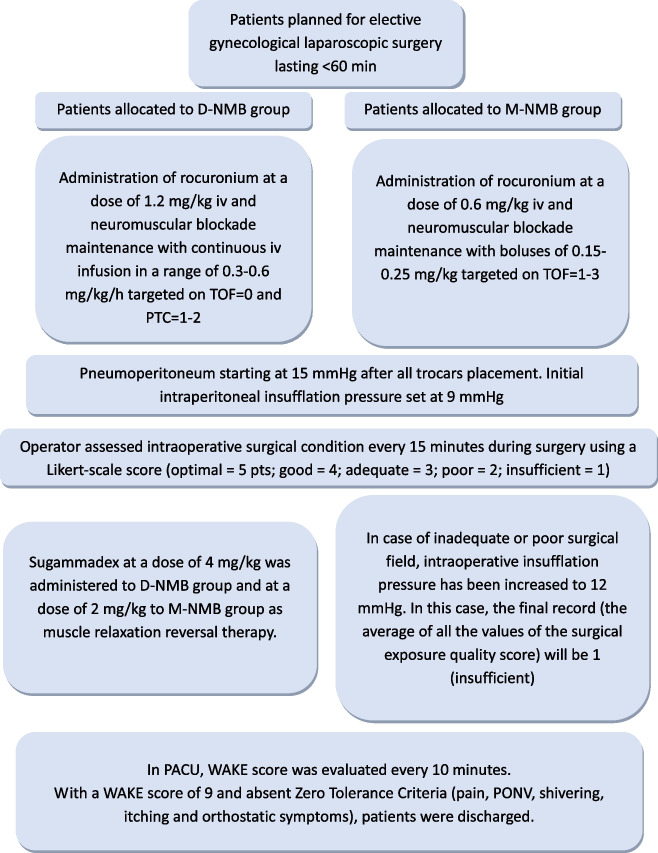
Study flowchart

In PACU, WAKE© score was evaluated every 5 min. When WAKE© score was higher than 9 and Zero Tolerance Criteria (pain, PONV, chills, itching, and orthostatic symptoms) were absent, patients were discharged (Fig. 1) from PACU.

Post-operative pain control strategy included diclofenac iv (50 mg), administered 12 h after first bolus and paracetamol iv (1000 mg) every 6 h started immediately after surgery. Tramadol iv (1 mg/kg) was considered as rescue therapy for acute post-operative pain.

### Statistical analysis

Data were expressed as means with standard deviation or as numbers with percentages. Univariate comparisons of dichotomous data were performed using the *χ*^2^ test with continuity correction. Comparisons between groups were performed using *t* test to evaluate group means by assuming equal within-group variances. The primary analysis was an intention-to-treat comparison of random assigned treatments. The effect of D-NMB on the primary, secondary and tertiary outcomes was quantified as mean difference (MD) with 95% confidence interval (CI). A 2-sided *P* < 0.05 was considered statistically significant. Statistical analysis was performed using SPSS version 19.0 (IBM Inc.).

## Results

**Fig. 2:**
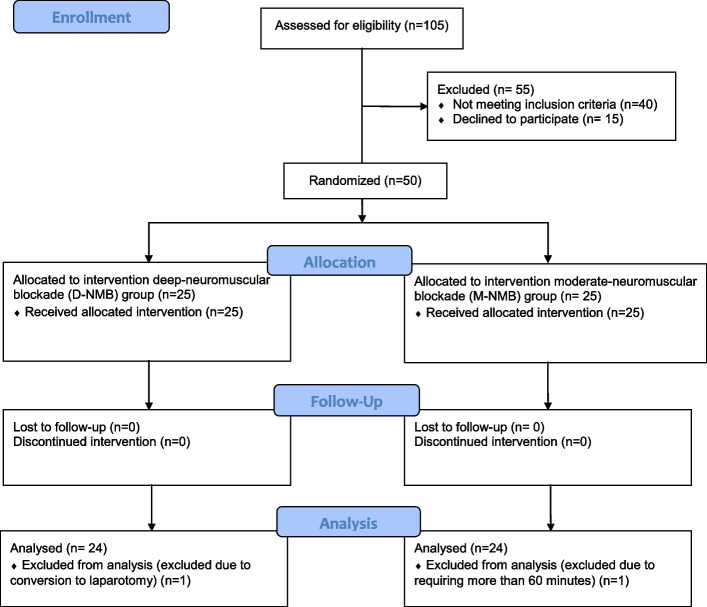
CONSORT flow diagram

One hundred five patients were assessed for eligibility: 55 were excluded (40 patients didn’t meet inclusion criteria, 15 patients declined to participate); 50 patients, who underwent elective gynecological laparoscopic surgery, met the inclusion criteria and were enrolled in the trial. One participant in each group was excluded after randomization and was not included in the analysis (Fig. 2): the one assigned to the D-NMB group was excluded due to conversion to laparotomy while the one assigned to M-NMB group was excluded due to surgery longer than 60 min. At 60 min, the measurements were available only for 8 patients in D-NMB group and 15 in M-NMB group.

Table 1 shows demographic characteristics of the included patients.

**Table 1:** Characteristics of patients

	Deep NMB (*n* = 24)	Moderate NMB (*n* = 24)	*p* value
Age (years)	39 ± 3	41 ± 3	0.2
BMI (kg/m^2^)	24 ± 3	23 ± 3	0.6
ASA physical status			0.3
1	6 (25%)	3 (12%)	
2	18 (75%)	21 (87%)	
Comorbidities			0.8
None	14 (58%)	16 (66%)	
Cardiovascular	4 (16%)	4 (16%)	
Thyroid dysfunction	3 (12%)	3 (12%)	
Allergies	3 (12%)	1 (4%)	
Gynecologic disease			0.1
Ovarian disease	9 (37%)	15 (62 %)	
Sterility treatment	10 (41%)	4 (16%)	
Endometriosis	5 (20%)	5 (20%)	

Values are mean, standard deviation (SD) or number of patients (proportion, %

*ASA* American society of anesthesiologists, *BMI* body mass index, *NMB* neuromuscular block

Boldface data, statistically significant

The mean BMI was about 24 in D-NMB group and 23 in M-NMB group; the average age of patients was 39 years for D-NMB group and 41 for M-NMB group.

The average of intra-operative global surgical condition was 4 for the D-NMB group and 3 for the M-NMB group (*p* value < 0.01). It is the mean of the global evaluation of the operative field made by the surgeon at the end of each intervention.

The mean intraperitoneal insufflation pressure was lower in the D-NMB group than in the M-NMB group (8 vs 10 mmHg) with a significantly higher overall surgical site exposure assessment in the D-NMB group than in the M-NMB group.

Trendelenburg position proved to be necessary for all patients (Table 2). We highlighted an average of 17° for the M-NMB group and 13 for the D-NMB group (*p* value < 0.01).

**Table 2:** Intra-operative and post-operative details

	Deep NMB (*n* = 24)	Moderate NMB (*n* = 24)	*p* value
Intra-operative surgical complications			1
None	21 (87%)	21 (87%)	
Intra-operative bleeding	3 (12%)	3 (12%)	
Patients discharged from operating room (n.o)			0.1
At 15–30 min	6 (25%)	3 (12%)	
At 30–45 min	10 (42%)	6 (25%)	
At 45–60 min	8 (33%)	15 (63%)	
Patients requiring rescue analgesics in PACU (n.o)	8 (33%)	15 (63%)	**0.04**
Patients requiring an additional dose of sugammadex (n.o)	0 (0%)	0 (0%)	1
Trendelenburg (degrees)	13 ± 1	17 ± 2	**< 0.01**
Patients with symptoms in PACU at 15 min (n.o)			0.07
Pain	12 (50%)	18 (75%)	
PONV	0 (0%)	0 (0%)	
Itch	0 (0%)	0 (0%)	
Patients with PONV at 24 h (n.o)	2 (8%)	0 (0%)	0.1
Patients with respiratory complications at 24 h (n.o)	0 (0%)	0 (0%)	1
Mean VAS at 24 h	0.2 ± 1	1 ± 1	**< 0.01**

Values are mean, standard deviation (SD) or number of patients (proportion, %)

*n.o* number, *NMB* neuromuscular block, *PACU* post-anesthesia care unit, *PONV* post-operative nausea and vomiting, *VAS* visual analogue scale for pain

Boldface data, statistically significant*not predictable, constant

Peak airway pressure after CO_2_ insufflation was less in D-NMB group than in M-NMB group (*p* value < 0.01).

Intraoperative anesthetic complications were less in the D-NMB group than in the M-NMB group (4% vs 38%) with a significantly higher hemodynamic instability in the M-NMB group (33% vs 4%).

PONV and itching were absent in both groups.

Neither patient in either group required an additional dose of sugammadex in the postoperative period.

Mean Visual Analogue Scale (VAS) was 0.2 for D-NMB group and 1 for M-NMB group (*p* value < 0.01); percentage of patients with post-operative pain was significantly lower in the D-NMB group (50%) than in the M-NMB group (75%) (*p* value 0.07) at 15 min in PACU as well as after 24 h from surgery.

The average length of stay in PACU was 14 min for the D-NMB group and 22 min for the M-NMB group (*p* value 0.02) (Table 3).

**Table 3:** Outcomes

	Deep NMB (*n* = 24)	Moderate NMB (*n* = 24)	*p* value
Intra-operative surgical condition			
At 15 min			
Extremely poor	12%	12%	0.2
Poor	0%	8%	
Acceptable	0%	20%	
Good	18%	25%	
Optimal	70%	35%	
At 30 min			
Extremely poor	0%	10%	0.1
Poor	0%	8%	
Acceptable	4%	12%	
Good	12%	37%	
Optimal	84%	33%	
At 45 min			
Extremely poor	0%	0%	0.2
Poor	0%	0%	
Acceptable	4%	12%	
Good	18%	50%	
Optimal	78%	38%	
At 60 min			
Extremely poor	0%	0%	0.4
Poor	0%	0%	
Acceptable	0%	7%	
Good	39%	38%	
Optimal	61%	55%	
Intra-operative global surgical condition (mean)	4 ± 1	3 ± 2	**< 0.01**
Mean intraperitoneal insufflation pressure (mmHg)	8 ± 1	10 ± 1	**< 0.01**
Peak airway pressure (mmHg)	22 ± 3	25 ± 4	**< 0.01**
Time in PACU	13 ± 8	22 ± 10	**0.02**
Intra-operative anesthetic complications			0.2
None	23 (95%)	15 (62%)	
Hemodynamic instability	1 (5%)	8 (33%)	
Difficult intubation	0 (0%)	1 (5%)	

Values are mean, standard deviation (SD) or number of patients (proportion, %)

*NMB* neuromuscular block, *PACU* post-anesthesia care unit

Boldface data, statistically significant

None had respiratory complications in the postoperative period and in the first 24 h.

## Discussion

### Main findings

This study aimed to evaluate the superiority of D-NMB over M-NMB in gynecological laparoscopic surgery.

The trial showed that D-NMB was associated with improved surgical condition, less stay in PACU and less hemodynamic instability. The difference in average scores in the various time intervals (15, 30, 45, and 60 min) is not statistically significant.

### Implication

Muscle relaxants are used daily for laparoscopic surgery for their many intraoperative advantages.

A deep neuromuscular block allows better visibility of the operating field, patient immobility, lower insufflation pressure and consequently less wall stretch.

This latter factor is particularly important for the intra and post-operative analgesic implications.

Lower intraperitoneal pressure during surgery minimizes inflammatory response which results in a lower impact on post-operative period both in terms of hospital stay and postoperative pain.

A deep neuromuscular block also allows better mechanical ventilation and guarantees a better adaptation of the patient to pneumoperitoneum without affecting hemodynamics.

On the other hand, muscle relaxants can lead to well-known adverse post-operative effects such as post-operative residual curarization (PORC) [7] or incomplete recovery of upper respiratory functions [8, 9].

For this reason, a close neuromuscular monitoring (acceleromyography or TOF) is likely mandatory during surgery as well as the availability of a rapid and selective reversal like sugammadex which allows a complete and effective antagonization [10–12] effect of rocuronium [11, 13, 14] without prolonging the length of stay in PACU.

Outpatient surgery or day surgery can reduce the cost of hospitalization, optimize institutional resources [15] and provide healthcare services at high quality standards with safety and efficacy.

Our findings emphasize the benefits of using deep neuromuscular block compared to M-NMB.

### Limitations

This trial has several limitations.

Firstly, it is a single-center and slightly generalizable study.

Secondly, it does not evaluate the medium- and long-term effects of deep neuromuscular block on post-operative implications (e.g., post-operative pain at 3, 6, 12, 24, and 36 h after surgery, time for resumed intestinal canalization, time for patient mobilization).

Moreover, patients’ demographic characteristics and comorbidities are not completely balanced between the two groups.

## Conclusions

Deep neuromuscular block guarantees better intraoperative surgical condition and fewer postoperative complications during gynecological laparoscopic surgery as demonstrated by this trial.

The surgical team evaluated surgical field with deeper muscle relaxation as the best.

The rate of intraoperative hemodynamic and respiratory complications (hemodynamic instability and peak airway pressure) was lower in the deep block group.

VAS, PONV, itching, and time to discharge from PACU were lower in the experimental group than in the control group.

Therefore, deep neuromuscular block optimizes intra- and post-operative conditions and improves patients’ comfort.

Future large, multicenter trials are needed to confirm our findings.

## Data Availability

Consent for publication of raw data was not obtained but dataset is fully anonymous in such a manner that it can be easily verified by any user of the dataset.
